# Supplementary material for: Learning curve and surgical outcome of robotic assisted colorectal surgery with ERAS program
Source: Sci Rep. 2022 Nov 29;12:20566. doi: 10.1038/s41598-022-24665-w (PMC9709162; doi:10.1038/s41598-022-24665-w)
Supplement: Supplementary file 1 — Supplementary Information 1. [file 41598_2022_24665_MOESM1_ESM.zip › LSC Raw data-Ñ[▒K/134/H_ñw╝╨░O▒Kñσ.pdf]

正式摘要(電子簽章：000000224878196)		
W83 / W83 ☐ 英文標題(注意:無英文姓名-請補病人基本檔!)	☐【讀取】憑證	

臺中榮民總醫院

出院病歷摘要

轉入號 : 轉出號 :

[redacted]

(8)流水編號	
(9)入院日期	110/08/24 (2021) 直腸外科 W83 - 072
(10)轉科日期	
(11)出院日期	110/09/06 (2021) 住院天數計 13 日

診斷

- 入院 :1. Colon tumor at hepatic flexure with regional lymph node involvement and liver metastasis, cT2N2aM1a
- 出院 :1. Colon tumor at hepatic flexure with regional lymph node involvement and liver metastasis, cT2N2aM1a
  -s/p right hemicolectomy (laparoscopy conversion to open) on 2021/08/26, pT4bN2bM1a

主訴

- Body weight loss aabout 5 kg within 2-3 months

病史

- This 72-year-old woman denied systemic disease before.
  This time, she had chronic constipation and accompany with body weight loss about 5kg within recent 2-3 months. Due to those, she went to 長安H. for help.colonscope and panendoscope was done. colon tumor at hepatic flexure was noted during colonscope examination. Lymph node involvement and liver metastasis cT2N2aM1a. She had also mentioned palpable neck lymph node recently. As above problem, she went to our OPD for help. Under impression of colon cancer with lymph node and liver metastasis, she was admitted for further surgical intervention.

體檢發現

-
  - General appearance :
    - easy looking , conscious clear , without cardiopulmonary distress
    - Vital signs自動帶入date 體溫(BT) 脈搏(P) 呼吸(R) 血壓(BP) 身高(BH) 體重(BW) BMI
      20210824 35.9℃ 61次/分 18次/分 105/51mmHg 152.1cm 41kg 17.7
    - Vital signs: B.P. 105/51 mmHg , P.R. 61 /min , R.R. 18 /min , B.T. 35.9 ℃
  - HEENT :
    - Eyes: no anemic conjunctiva, no icteric sclera, normal EOM
    - ENT: no otorrhea, no rhinorrhea, no erythematous mucosa, no injected tonsil, no deformity
  - Neck: supple, freely movable, trachea midline,no deformity, no engorged jugular vein , no palpable thyroid or lymph node enlargement
  - Chest:
    - Inspection: Grossly normal, symmetric and fully expansion, no accessory muscle used, no operation scar, no spider angiomata, no gynecomastia
    - Palpation: no palpable mass or nodule, no subcutaneous emphysema, no tenderness
    - Ausculation: clear breathing sound
  - Heart:
    - Percussion: normal heart size
    - Ausculation: regular heart beat, normal S1, S2, no gallop, no murmur
  - Abdomen:
    - Inspection: in
      - flat contour, symetrically, no scar, no stria, no dilated veins, no rashes
    - Ausculation: normoactive bowel sound, no abdominal bruits
    - palpation: soft, no muscle guarding or rigidity, no local tenderness, no rebounding pain, no palpable mass, no palpable pulsation

- o Percusssion: no shifting dulllness, no hepatomegaly, no splenomagaly
-

Peripheral pulsation :	CA	BA	RA	FA	PA	PTA	DPA
Right side :	++	++	++	++	++	++	++/+++
Left side :	++	++	++	++	++	++	++/+++

- Genito-anus: no tenderness or palpable mass lesion or rectal shelf, normal external genitalia, normal anal tone, brown stool

CRS CONDITION

-
  - Lung

手術日期及方法

- 手術日期及方法20210826 1. Laparoscopy convert to open due to advanced tumor, Right hemicolectomy with side-to-side anastomosis, Barcelona method with GIA 75mm*2, re-inforcement with silk (+).
  2. Vessel ligation : ileo-colic, right colic, and right branch of middle colic vessels
  3. Repair of mesentery defect: nil.
  4. cholecystectomy was done due to tumor severe adhision to Gall bladder
  5. Surgiwrap +
- 手術結果及發現：
  - 1.One hudge tumor, measured about 8 x 10 cm in size, located at hepatic flexture colon, which invaded to Gall bladder 2.No other gross tumor or polyp was found. 3.There was no visible liver metastasis or visible peritoneal seeding. 4.The duodenum was intact. 5.Some cloudy whitish ascited noted

住院治療經過

- After admission, pre-op survey was done. Right colectomy (lapa converted to open) with side to side ileocolonal anastomosis were smoothly performed on 2021/08/26. After surgery, post-op care was applied. Wound pain was tolerable. Symptomatic treatment was given. Antibiotics with Cefazolin 1000mg Q8H + Metronidazole 500mg Q6H (8/29-9/5) was prescribed for prophylaxis. She started to try semi-liquid since 09/01. She complained about left lower limbs swelling, while abdominal CT showed no DVT, lymph node metastasis couldn't be ruled out. There was much improvement under flexible socks use. Under her relative stable condition, she was discharged on 09/06.

合併症

- Nil

一般檢查

- 累積報告：

DATE	TIME	ALB	TP	BIL.T	BIL.D	ALKP	AST	ALT	LDH	AMY	LIPASE	NH3	Ferritin	UA
1100826	1632	3.0		0.24	0.09	81	24	17						
1100830	0912	2.7		0.28	0.09		48	41						
1100902	0844	3.1		0.38	0.12		58	20						

DATE	TIME	WBC	RBC	HGB	HCT	MCV	PLT	BLST	NEUT	BAND	SEG	LYM	MON	EOS	BAS
1100826	1632	8970	4.30	12.5	38.1	88.6	335		88.9			6.2	4.3	0.4	0.2
1100830	0919	8560		13.4	39.4		350		85.3			8.2	5.7	0.7	0.1
1100902	1057	3520	3.84	11.5	33.5	87.2	291		66.4			13.9	15.1	4.3	0.3

DATE	TIME	NA	K	CL	BUN	CREAT	eGFR	CA	Mg	P	CRP	ESR	PCT	D-Dimer	LACTATE
1100826	1632	138	3.7	106	10	0.62	100.57	7.7							
1100830	0906														14.2
1100830	0912	133	4.1		16	0.52	123.2	7.6							
1100902	0844	136	3.7		14	0.55	115.48	7.6							
1100902	1052													4.39	

DATE	TIME	GLUCOSE	HbA1C	CHOL	TG	HDL	LDL	KETONE	CK	CKMB	TRO-T	PT-P	APTT-P	NT-pro BNP
1100826	1632	181												

- 放射線報告：**檢驗項目 :CT,Peripheral vascular CTA 報告日期 :1100906**
  主　訴：
  Result:N
  Finding:
  Contrast-enhanced CT scan of bilateral lower limbs with indirect CT venogram shows:
  > A case for r/o DVT
  > Patent of venogram of bilateral lower limbs
  > Atherosclerotic calcificaitons over visible abdominal aorta & its major branches
  > Mild ascites, pneumoperitoneum are noted, may be due to post OP chang
  > S/p cholecystectomy
  > Enalrged lymph nodes over para-aortic region, may be related to colon cancer.
  > Mild air bubble within UB, previous foley insertion is first considered.
  Impression:

- Patent of venogram of bilateral lower limbs

特殊檢查

病理報告

- 活體組織切片病理報告 手術日期：**110/08/26** 病理號：**S1026714**

診斷醫師:陳律臻 報告日期:110/09/03
Pathologic diagnosis:
Colon, terminal ileum, appendix and gallbladder, right hemicolectomy + cholecystectomy --- Adenocarcinoma, moderately differentiated, of ascending colon. Tumor invaded through serosa and involved the soft tissue of gallbladder and metastasized to mesocolic lymph nodes (8/22). The appendix, ileocecal valve, bilateral cut ends, gallbladder and liver are free of tumor.
Ancillary study for diagnosis:
IHC stains for PMS2 and MSH6 are done on section K. All above markers are preserved.
Prognostic and predictive factor:
1. Tumor size: 3.5x3 cm.
2. Depth of invasion: Tumor directly invades adjacent structures (specify: soft tissue of gallbladder).
3. Histologic grade: G2: Moderately differentiated.
4. Angiolymphatic invasion: Present, extensive.
5. Perineural invasion: Not identified.
6. Tumor deposits (discontinuous extramural extension): Present, specify number of deposits: multiple.
7. Macroscopic tumor perforation: Not identified.
8. Surgical Margins: a) Proximal Margin: Uninvolved by invasive carcinoma, distance of tumor from margin: 11 cm. b) Distal Margin: Uninvolved by invasive carcinoma, distance of tumor from margin: 6 cm. c) Radial or Mesenteric Margin: Uninvolved by invasive carcinoma, distance of tumor from margin: 5 mm.
9. Lymph node status: Involved (8/22).
10. Extranodal involvement: Present.
11. Tumor regression grading S/P CCRT: No known presurgical therapy.
12. Type of polyp in which invasive carcinoma arose: Not identified.
13. Additional pathologic findings: Not identified.
14. Pathological TNM Stage: pT4bN2b(According to the eighth edition, American Joint Committee on Cancer Staging Guidelines for Tumors).
15. TNM descriptors: Not applicable.
Gross description:
The specimen consists of a segment of colon, 14 cm in length, and 9 cm in proximal circumference, 6 cm in distal cut end circumference, and a segment of terminal ileum, 7 cm in length, and 4.5 cm in cut end circumference. An unremarkable appendix, 3x0.6x0.5 cm, is noted. A gray tan firm annular elevated and centrally ulcerative tumor, 3.5x3 cm, is noted, 6 cm to the distal cut end. An unopened gallbladder, 5x3x0.7 cm, is noted adhered to the mesocolic soft tissue. On cross section, the tumor invaded to the serosa and involved the soft tissue of gallbladder. The appendix, ileocecal valve, and both cut ends are free of tumor. Representative part for section: A) proximal cut end B) distal cut end C) ileocecal valve D) appendix E) cut end of gallbladder F-K) tumor L-Q) lymph nodes.
Note:
The interpretation of MMR panel and recommended studies are as below: (1) No loss of nuclear expression of MMR proteins: low probability of microsatellite instability-high (MSI-H). (2) Loss of nuclear expression of MLH1 and PMS2: testing for methylation of the MLH1 promoter and/or mutation of BRAF is indicated (the presence of a BRAF V600E mutation and/or MLH1 methylation suggests that the tumor is sporadic and germline evaluation is probably not indicated; absence of both MLH1 methylation and of BRAF V600E mutation suggests the possibility of Lynch syndrome, and sequencing and/or large deletion/duplication testing of germline MLH1 may be indicated). (3) Loss of nuclear expression of MSH2 and MSH6: high probability of Lynch syndrome (sequencing and/or large deletion/duplication testing of germline MSH2 may be indicated, and, if negative, sequencing and/or large deletion/duplication testing of germline MSH6 may be

- indicated). (4) Loss of nuclear expression of MSH6 only: high probability of Lynch syndrome (sequencing and/or large deletion/duplication testing of germline MSH6 may be indicated). (5) Loss of nuclear expression of PMS2 only: high probability of Lynch syndrome (sequencing and/or large deletion/duplication testing of germline PMS2 may be indicated). (6) Please note: There are exceptions to the above IHC interpretations. These results should not be considered in isolation, and clinical correlation with genetic counseling is recommended to assess the need for germline testing.
  #T-59420_2 #M-81403_2 2 1105 S0000F
  #T-C4560_2 #M-81406_2 0 1105 000000
  #T-59200_2 #M-09183_2 0 1105 000000
  #T-63000_2 #M-09413_2 0 1100 000000

其它

出院時情況

- 3.改門診治療

出院指示

藥名	劑量	劑量單位	用法	頻次	數量	數量單位	現況	天數	單位
$Lederscon tab	1	TAB	PO	QID	32	TAB	TH	8	天
Mag. Oxide tab	1	TAB	PO	TID	24	TAB	TH	8	天
Pantoloc tab 40mg	1	TAB	PO	QDAC	8	TAB	TH	8	天
Aspirin tab 100mg	1	TAB	PO	QD	8	TAB	TH	8	天
TramaCET tab	1	TAB	PO	Q8HPRN	24	TAB	TH	8	天
Artelac eye drops		BOT	OU	TID	0	BOT	TH	1	瓶

出院計劃

- 門診日期
  - date on 2021/09/14
- check All-RAS gene test

主治醫師 2062F 蔣鋒帆 / 住院醫師 2336L 洪浚嚴

正式摘要(電子簽章：**000000224878196)**

W83 / W83

doctorRank:B
